# Repurposing the Tyrosine Kinase Inhibitors Targeting FGFR and VEGFR Pathways for Cancer Therapy: A Comprehensive Review

**DOI:** 10.3390/cancers17203354

**Published:** 2025-10-17

**Authors:** Sergei Boichuk, Tatyana Gessel

**Affiliations:** 1 Department of Pathology, Kazan State Medical University, 420012 Kazan, Russia; ivoilova.tatyana@mail.ru; 2 Department of Radiotherapy and Radiology, Faculty of Surgery, Russian Medical Academy of Continuous Professional Education, 125993 Moscow, Russia; 3 Central Research Laboratory, Kazan State Medical University, 420012 Kazan, Russia

**Keywords:** receptor tyrosine kinase inhibitors (RTKIs), FGFR and VEGFR pathways, cancer chemoresistance, ABC transporters, DNA damage repair (DDR) pathways, cancer stem cells (CSCs)

## Abstract

**Simple Summary:**

Tyrosine kinase inhibitors (TKIs) targeting the FGFR and VEGFR pathways, which exhibit potent angiogenic activities, are currently used in the therapy of a broad spectrum of human malignancies. Their efficacy is based on the selective targeting of overactivated signaling cascades in cancer cells and thereby inhibiting their proliferation and survival. Besides this specific modality, TKIs have also been reported to exhibit multiple “off-target” effects by interacting with ABC transporters and inhibiting their function. Additionally, TKIs can regulate the efficacy of diverse DNA damage repair (DDR) pathways, epithelial-to-mesenchymal transition (EMT), and the population of cancer stem cells (CSCs). Overall, the aforementioned “off-target” effects of TKIs are considered attractive tools for sensitizing cancer cells to conventional chemotherapies and overcoming cancer resistance to these therapies, thereby leading to more effective anti-cancer therapy with fewer adverse effects.

**Abstract:**

Resistance to conventional anti-tumor drugs is one of the significant challenges in oncology, responsible for treatment failure and patient death. Introduction of the targeted drugs (e.g., small molecule tyrosine kinase inhibitors (TKIs) and monoclonal antibodies) in cancer therapy significantly improved overall survival (OS) and progression-free survival (PFS) rates for selected groups of cancer patients and delayed the progression of advanced forms of human malignancies. However, the development of secondary resistance to the targeted drugs remains an unbeatable obstacle to a successful outcome in the long run, thereby making prognosis unfavorable for cancer patients with advanced, recurrent, and metastatic forms of disease. The review focuses on several mechanisms that regulate cancer resistance to conventional chemotherapies. This includes the upregulation of main types of ABC transporters (e.g., ABCB1, ABCC1, and ABCG2), which provides the efflux of chemotherapeutic agents from cancer cells. Additionally, the activation of diverse DNA damage repair (DDR) pathways, epithelial-to-mesenchymal transition (EMT), and the population of cancer stem cells (CSCs) are also discussed in detail, thereby illustrating the diverse molecular mechanisms of cancer sensitivity to chemotherapies. Recently, several TKIs, including those that were initially developed to specifically target FGFR and VEGFR pathways, have also been reported to exhibit “off-target” effects by interacting with ABC transporters and inhibiting their function. This, in turn, illustrates their potency in retaining chemotherapeutic agents within cancer cells and possessing a chemosensitizing function. Of note, FGFR and VEGFR inhibitors may behave as inhibitors or substrates of ABC transporters, depending on the expression of specific pumps and affinity for them, concentrations, and types of co-administered agents, thereby disclosing the complexity of this scenario. Additionally, the aforementioned RTKI can interfere with the other molecular mechanisms regulating tumor sensitivity to conventional chemotherapies, including the regulation of diverse DDR pathways, EMT, and the population of CSCs. Thereby, the aforementioned “off-target” functions of FGFR and VEGFR inhibitors can open novel approaches towards anti-cancer therapies and strategies aimed at counteracting cancer multidrug resistance (MDR), which is important especially as second- or third-line treatments in patients who have progressed on modern chemotherapeutic regimens. Notably, the strategy of using TKIs to potentiate the clinical efficacy of chemotherapies can extend beyond inhibitors of FGFR and VEGFR signaling pathways, thereby providing a rationale for repurposing existing TKIs as an attractive therapeutic approach to overcome cancer chemoresistance.

## 1. Introduction

Despite a broad spectrum of anti-tumor chemotherapeutic agents being introduced into clinical oncology over the past decades, the progression-free survival (PFS) and overall survival (OS) rates for cancer patients diagnosed with advanced, metastatic, and recurrent forms of disease remain unfavorable. Indeed, despite the initial effectiveness of specific chemotherapeutic agents, clinical responses over time tend to decline, reflecting the development of cancer resistance. This, together with the enormous numbers of systemic toxic effects of chemotherapy, makes the clinical situation worse and highlights an urgent need to develop novel therapeutic strategies overcoming cancer chemoresistance and decreasing the incidence and number of the systemic toxic effects of the conventional chemotherapies. It is well known that cancer cells can acquire resistance to chemotherapies via diverse molecular mechanisms, including the inhibition of programmed cell death [1], activation of DNA damage repair (DDR) mechanisms [2], the changes in the drug’s metabolism [3], aberrant activation of tyrosine kinases [4], and enhancing its excretion through ATP-dependent transport proteins, ABC transporters [5,6], etc. Given the numerous original articles and fundamental reviews that describe in detail the diverse molecular mechanisms of tumor resistance to conventional chemotherapies, this review does not aim to provide a detailed analysis of the myriad aspects of tumor resistance to chemotherapies. The specific mechanisms of tumor resistance to chemotherapeutic drugs that TKIs can modulate via providing their “off-target” effects are shown below.

## 2. Cancer Stem Cells (CSCs) and Chemoresistance

**Figure 1:**
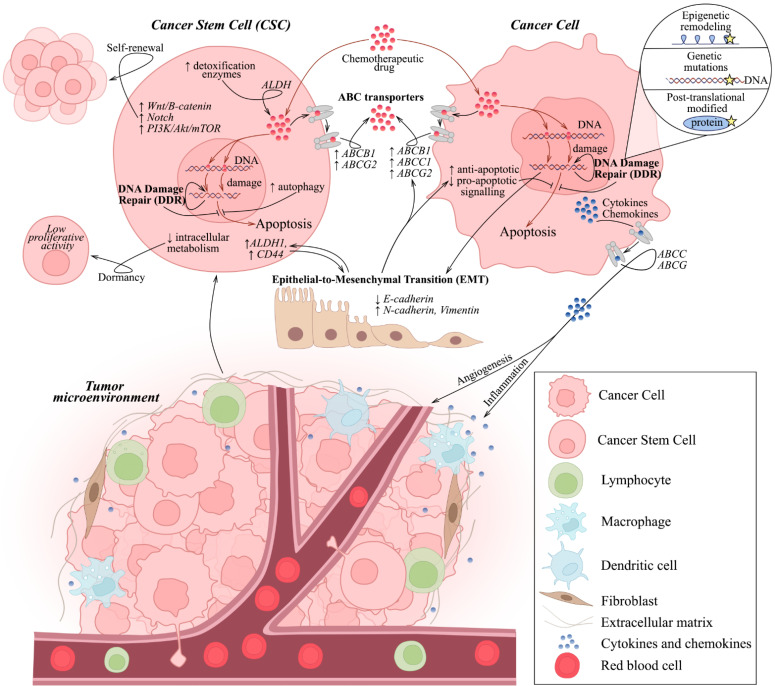
Crosstalk between DNA damage repair (DDR) pathways, ABC transporters, Epithelial-to-Mesenchymal Transition (EMT), and cancer stem cells (CSCs) in carcinogenesis, cancer progression, and development of multidrug resistance (MDR) of cancer. ↑ : increased expression/activity; ↓ : decreased expression/activity; ★: genomic and post-translational modifications.

CSCs possess multiple intrinsic and extrinsic properties, making them naturally resistant to chemo- and radiotherapy. As shown in Figure 1, this includes (a) enhanced DNA damage repair (DDR) mechanisms [7,8,9,10,11,12]; (b) overexpression of ABC transporters effluxing chemotherapeutic agents from cells and thereby minimizing DNA damage [13,14,15]; (c) enhancement of anti-apoptotic and pro-survival signaling pathways, including upregulation of anti-apoptotic proteins (i.e., BcL-2, Bcl-xl, cellular FLICE inhibitory protein (c-FLIP), inhibitor of apoptotic proteins (IAPs), etc.), deficiency of mitochondrial-mediated apoptosis [16,17,18] and overactivation of Wnt/B-catenin, Notch-, PI3K/Akt/mTOR-mediated cascades promoting self-renewal of CSCs [19,20,21]; (d) increased expression of detoxification enzymes, including aldehyde dehydrogenase (ALDH), inactivating chemotherapeutic agents [22,23,24]; (e) formation of a specific microenvironment (niche), including interaction with stromal cells and extracellular matrix, modulating sensitivity to specific chemotherapeutic agents [25,26,27,28]; (f) higher levels of autophagy, known as an adaptive mechanism under stressful situations, including exposure to DNA-damaging factors [29,30,31]; (g) dormancy of CSCs rendering them non-sensitive to DNA-damaging therapy targeting predominantly the cells with high proliferative rate; (h) close relationship between EMT in cancer cells and their SC-like phenotype [32,33].

Therefore, targeting the pathways mentioned above could effectively resensitize CSCs to chemo/radiotherapy and eradicate the rapidly dividing bulk tumor cell population from CSCs, which might be critical for improving the efficacy of anti-cancer therapies. In particular, combination strategies targeting DDR proteins simultaneously and inducing DNA damage could help overcome the cytoprotective function of activated DDR in CSCs. These aspects were described in detail in several fundamental reviews, as indicated below [34,35].

## 3. DNA Damage Repair (DDR) and Sensitivity of Cancer to Chemotherapy

Given the pivotal roles of genetic integrity in cell survival and proliferation, chemotherapeutic DNA-damaging agents have been developed and widely used to induce DNA damage in rapidly proliferating tumor cells, thereby inducing replicative stress and cell death. Therefore, alterations in DDR pathways are well-known features of cancer cells, which enable them to adapt to the DNA damage caused by genotoxic chemotherapy and radiotherapy. As shown in Figure 1, this can be achieved by (a) genetic reversion of DDR deficiency in cancer cells, responsible for the initial tumor response to chemotherapy; (b) post-translational modifications of DDR proteins modulating DDR signal transduction, regulating DDR proteins stability and their dynamic phase separation; (c) epigenetic remodeling, including chromatin relaxation surrounding DNA damage sites and providing sufficient accumulation of DDR proteins in these sites. The best-known example of the first scenario is the use of poly (ADP-ribose) polymerase (PARP) inhibitors, which have demonstrated encouraging results for the treatment of BRCA-mutant (i.e., homology-recombination (HR)-deficient) ovarian and breast tumors. However, some BRCA-deficient tumors can rapidly acquire PARPi resistance, driven by the genetic restoration of BRCA protein function and, therefore, the reversal of HR activity [36]. Post-translational modifications of DDR proteins are well-established and regulate appropriate DNA damage signaling. In particular, phosphorylation of the DNA damage sensor kinases, ataxia telangiectasia mutated (ATM), ataxia telangiectasia mutated and Rad3-related (ATR), and DNA-dependent protein kinase catalytic subunit (DNA-PKcs), is a well-known signature of their activation and is also responsible for the phosphorylation of downstream DDR effector proteins and the magnitude of the DDR signaling cascade.

On the other hand, multiple reports demonstrate that cancer cell metabolites can also effectively trigger post-translational modifications of DDR proteins, thereby increasing their efficacy and promoting cancer resistance to DNA-damaging therapies. For example, lactate-induced lactylation of several DDR proteins involved in homology-mediated DNA repair, such as meiotic recombination 11 (MRE11) and Nijmengen breakage syndrome 1 (NBS1), has been recently shown to promote HR and contribute to cancer resistance to PARPi or cisplatin [37,38]. Lastly, epigenetic remodeling also contributes to cancer chemoresistance. In particular, this includes chromatin relaxation surrounding DNA damage sites, providing sufficient access for DDR proteins to these sites.

Therefore, an acquired resistance of cancer to DNA-damaging chemo- and radiotherapies resulting from dysregulated DDR activity might be a significant obstacle for successful chemo- and radiotherapies used in adjuvant settings. Thus, a combined therapy targeting specific DDR defects in case of chemotherapy-induced DNA damage represents a promising approach to overcome cancer resistance and sensitize the malignancies to conventional chemotherapies. Indeed, targeting of DNA damage sensor kinases ATM and ATR, coupled with chemotherapeutic drugs, increased their anti-tumor efficacy [39]. Similarly, inhibition of downstream DDR kinases, such as checkpoint kinase 1 (CHK1), potentiated the anti-cancer activities of chemotherapeutic agents [40], thereby illustrating that targeting of deregulated DDR proteins might be an effective strategy to reverse cancer drug resistance.

## 4. Epithelial-to-Mesenchymal Transition (EMT) and Cancer Resistance to Chemotherapies

EMT is a reprogramming process that converts epithelial cells into a more mesenchymal state, associated with the loss of adhesion between epithelial cells, thereby promoting their invasion and migration. Besides these well-known features mediating cancer progression, EMT might be associated with the acquisition of the tumor’s resistance to chemotherapies. This effect may be due to multiple mechanisms, including tight crosstalk between EMT and DNA repair mechanisms, which have been discussed in detail previously and play a crucial role in cancer’s sensitivity to DNA-damaging therapy. Indeed, multiple reports illustrate that EMT could be a result of unrepaired DNA lesions induced by a broad spectrum of DNA-damaging factors, including alkylating and microtubule-targeting agents [41,42,43]. This point was supported by the elegant data illustrating the re-expression of wild-type H2AX, but not a DNA-repair inactive phosphorylation site mutant, effectively reversing the EMT phenotype [44]. Moreover, several DDR proteins antagonize EMT and vice versa. For example, BRCA1 inhibits EMT transcription factors TWIST1 and FOXC1/2 [45], and RPA80, the upstream adapter molecule of BRCA1, effectively suppresses *ZEB1* expression [46]. In contrast, SNAIL and SLUG suppress BRCA1 by repressing its promoter or by recruiting the chromatin demethylase LSD1 [47]. Next, EMT is associated with an increased expression of ABC transporters (e.g., ABCB1, ABCG2), which pump chemotherapeutic agents out of the cancer cells [48]. Besides these factors, several EMT transcriptional factors (e.g., *Snail*, *Twist*, and *Zeb1*) have been shown to upregulate CSC markers, including ALDH1 and CD44, thereby promoting cancer chemoresistance [49,50,51]. Lastly, EMT transcriptional factors are also known as potent inhibitors of pro-apoptotic genes (e.g., *BIM*, *PUMA*) and upregulate anti-apoptotic proteins (e.g., Bcl-2), thereby conferring cancer resistance to DNA-damaging factors.

Therefore, reversing and/or reducing EMT phenotype in cancer is also considered a promising strategy to overcome cancer resistance to chemotherapies, as described in detail in several fundamental reviews [52,53,54].

## 5. ABC Transporters and Chemoresistance

ATP-binding cassette (ABC) transporters are ubiquitous transmembrane proteins involved in ATP-dependent translocation of a broad spectrum of substances across membranes. The superfamily of ABC transporters comprises 48 proteins, organized into seven distinct families, exhibiting a wide range of substrate specificities and functionalities. In particular, ABC transporters are responsible for multidrug resistance (MDR) in microbes and cancer cells due to the excessive efflux of therapeutic agents from these cells, thereby contributing to the failure of antibacterial therapy and chemotherapy [55,56]. The transport cycle of these proteins is associated with a switch in protein conformation from open to the inside of the cell to open to the extracellular space, which is mediated by the sequential attachment of the substrate in the substrate-binding site of the transmembrane domain, ATP in the nucleotide-binding domain of the protein, and its subsequent hydrolysis to ADP [57,58]. P-glycoprotein/multidrug resistance protein 1 (P-gp/MDR1/ABCB1), multidrug resistance-associated protein 1 (MRP1/ABCC1), and breast cancer resistance protein (BCRP/ABCG2) are the most significant ABC transporters clinically proven to be associated with multidrug resistance during cancer therapy. Although substantial structural and functional similarities exist between the proteins above, they exhibit different substrate specificities, resulting in an excessive efflux of a broad spectrum of toxic substances from malignant cells, which renders them resistant to DNA-damaging chemotherapies and reduces their effectiveness [56,59,60,61]. Besides an excessive efflux of chemotherapeutic agents from cancer cells, ABC transporters regulate other processes that may be indirectly involved in cancer progression and resistance to chemotherapies. These processes include the induction of metabolic changes in tumor cells (increased lipid metabolism) and the transport of pro- and anti-inflammatory cytokines and chemokines, thereby promoting angiogenesis and facilitating antigen processing and presentation. Overall, these factors contribute to the establishment of the specific tumor microenvironment, known as the “malignant niche” [61,62,63]. Thus, the role of ABC transporters in cancer development and progression is more complex and beyond the efflux of chemotherapies from malignant cells.

## 6. The Inhibitors of ABC Transporters

Given the crucial role of ABC superfamily proteins in transporting endogenous and exogenous substrates, including a wide range of chemotherapeutic agents, considerable efforts have been made to develop specific inhibitors that can modulate the functions of ABC transporters [60,61]. Such drugs would make it possible to sensitize cancer cells with intrinsic or acquired multidrug resistance to conventional chemotherapeutic drugs. Despite the significant success of developed inhibitors of ABC transporters in vitro, none of them has been approved for clinical use. However, research on the effects of these substances on ABC transporters and other mechanisms associated with the multidrug resistance development is still ongoing [60,64].

## 7. P-Glycoprotein/ABCB1 Inhibitors

The influence of verapamil, a first-generation inhibitor of P-gp, on these ABC transporters was discovered more than 40 years ago in lymphoid neoplasm P388 cells [65]. A considerable amount of evidence has accumulated, indicating increased intracellular accumulation of various P-gp substrates when used in combination with the described calcium channel blocker and its analogues in cell lines, as well as tumor cells from patients [65,66,67]. The transport mechanism of verapamil by P-gp was revealed by using molecular dynamics simulation, and similarities with the process of doxorubicin transfer were shown despite differences in relative amino acid residues [68]. Unfortunately, dose-limited side effects from the cardiovascular system were observed in patients with chemoresistant B-cell neoplasms [66]. At the same time, conflicting results have been reported regarding the impact of verapamil on other ABC transporters, specifically ABCC1 (MRP1) and ABCG2 (BCRP). On the one hand, verapamil stimulated glutathione efflux mediated by MRP1, leading to apoptotic cell death [69]. On the other hand, verapamil had no influence on ABCG2 in BCRP-overexpressing NCI-H460/MX20 cells [70].

Cyclosporin, a well-known immunosuppressive drug, demonstrated high potency to overcome chemoresistance of cancer cells due to its ability to bind with and inhibit the activity of ABCB1 [71]. This was due to its ability to act as a competitive inhibitor of the alkaloid-binding site of P-gp [72]. Besides ABCB1, the following studies demonstrated cyclosporin’s activity against the other types of ABC transporters, including ABCC1 and ABCG2 [73,74,75]. In contrast to ABCB1, cyclosporin exhibited non-competitive inhibitory activity against ABCG2 [76]. Although cyclosporin A has a high affinity for binding to ABC transporters, its addition to the combination of mitoxantrone and etoposide did not improve treatment outcomes for patients with refractory or relapsing acute myeloid leukemia (AML) who had developed resistance to these chemotherapies [77]. Moreover, the addition of cyclosporin A did not prolong the durations of remission or improve overall survival (OS) for children with AML, and its efficacy was independent of P-gp expression [78]. The attempts to improve the clinical effectiveness of cyclosporin A are currently ongoing. For example, several approaches have been designed to enhance the co-delivery of cyclosporin A and chemotherapeutic agents, including docetaxel, doxorubicin, and mitoxantrone to cancer cells [79,80,81]. The clinical benefits of these approaches remain to be further elucidated.

Second-generation P-gp inhibitors were designed through structural modifications of first-generation compounds, aiming to increase their specificity and reduce toxicity. For example, the activity of the R-enantiomer of verapamil, dexverapamil, was much less effective against the calcium channels. In contrast, its ability to target the ABCB1 protein remained unchanged compared to verapamil. This upgrade of verapamil improved its pharmacokinetics and clinical outcomes and reduced the cardiotoxicity of this calcium channel inhibitor [82,83]. Another second-generation inhibitor, valspodar (PSC-833), exhibited similar activity as its precursor, cyclosporine A, but acted exclusively on P-gp, with no effect on ABCC1 or ABCG2 [73]. However, the use of valspodar in combination with chemotherapy also did not show significant benefits for cancer patients in comparison with chemotherapy alone. It was also accompanied by systemic side effects, which required a reduction in the doses or discontinuation of therapy [84,85,86,87].

Therefore, third-generation ABC transporter inhibitors were subsequently developed, which had advantages over their previous analogues, including their selectivity and reduced number of side effects. The list of these inhibitors includes tariquidar, elacridar, and zosuquidar, known as non-selective inhibitors of ABC transporters, which act predominantly on ABCB1 and ABCG2 when used in high (micromolar) concentrations [70,88,89]. In contrast, in low nanomolar concentrations, they can serve as substrates for ABCB1 and ABCG2 [70,88]. Notably, tariquidar can function as an inhibitor of ABCB1 and as a substrate for ABCG2 [89]. Interestingly, to induce the conformational changes of the ABCB1 transporter leading to a loss of its functional activity, two molecules of tariquidar or three molecules of elacridar are required [88,90,91]. Based on its high affinity for ABCB1, tariquidar is currently recognized as a “gold standard” for ABCB1 inhibition. It is widely used in in vitro and in vivo studies to develop novel, effective drugs that overcome cancer chemoresistance by inhibiting the efflux of chemotherapeutic agents from cancer cells [92,93]. Similarly, elacridar, a third-generation P-gp inhibitor, effectively inhibited P-gp activity and reversed multidrug resistance in multiple cancer cell lines, thereby enhancing sensitivity to chemotherapeutics in vitro. In particular, this has been demonstrated for ovarian cancer cell lines using both 2D and 3D spheroid cultures [94], as well as non-small cell lung cancer (NSCLC) cell lines [95].

In addition to classical chemotherapeutic agents, third-generation ABC transporter inhibitors have also been demonstrated to be potent inhibitors that retain targeted drugs, including small-molecule tyrosine kinase inhibitors, within cancer cells. In particular, elacridar effectively potentiated the cytotoxic and anti-proliferative activities of imatinib mesylate against chronic myeloid leukemia (CML) cells overexpressing ABCB1 and ABCG2 [96]. Elacridar also potentiated the effect of sunitinib against renal carcinoma cells, resulting in an enhanced cytotoxic effect via the inhibition of P-gp activity [97].

After demonstrating the preclinical efficacy of third-generation ABC transporter inhibitors, early-phase clinical trials have shown their good tolerability with minimal side effects when used alone or in combination with chemotherapies. In particular, this was found for tariquidar in healthy individuals [98]. Similarly, phase I studies for elacridar demonstrated the minor side effects and good pharmacokinetic profiling [99]. In an early-phase trial, elacridar increased the plasma levels of oral paclitaxel by inhibiting intestinal P-gp activity [100]. A similar effect was observed for oral topotecan used in combination with elacridar, suggesting a potential use for this P-gp inhibitor in combination with oral chemotherapy for long-term treatment [101]. When combined with intravenous chemotherapy, including alkylating agents, elacridar demonstrated a high safety profile with relatively mild systemic side effects (the most common was neutropenia) in solid tumors [102].

However, despite the acceptable safety profile of the early phase trials, elacridar was not further developed in later clinical trials. In contrast, the ability of tariquidar to sensitize human malignancies to chemotherapies was a subject for detailed examination over a significantly longer period of time. A phase I study combining tariquidar with the microtubule-targeting drug vinorelbine also demonstrated good tolerability. Notably, the lack of pharmacokinetic interactions between the drugs above was found, which is a common issue for MDR1 inhibitors in clinical trials. Despite this, there was a lack of overall response (OOR) in this trial [103]. Similarly, low (<10%) rates of OOR were observed in more recent phase I trials, which combined tariquidar with docetaxel, vinorelbine, or doxorubicin for a broad spectrum of refractory solid tumors [104]. Low rates of OOR were also observed in the phase I trial, combining tariquidar with docetaxel for patients with lung, cervical, and ovarian cancer [105]. Of note, the expression of P-gp was not assessed for some of the cancer patients enrolled in the aforementioned clinical trials. Indeed, an improvement in ORR rates (up to 20%) was observed for cancer patients who exhibited positive P-gp staining before treatment [106].

Overall, despite the significant decrease in systemic toxicity, the clinical benefits of the combined use of tariquidar and chemotherapy remain controversial. Collectively, the small molecules specifically developed for P-gp inhibition were largely unsuccessful in clinical trials. Indeed, the first- and second-generation P-gp inhibitors were burdened with significant side-effect toxicities. In contrast, the third generation of P-gp inhibitors generally exhibited a much more acceptable safety profile, but they still demonstrated limited efficacy.

Based on these studies, natural compounds are also being actively studied to examine their potency in reversing the MDR phenotype in cancer cells and resensitizing human malignancies to certain chemotherapies. Therefore, the fourth generation of P-gp inhibitors is composed predominantly of natural compounds, including a variety of phytochemicals, such as flavonoids (e.g., quercetin, curcumin), terpenoids (e.g., guaiacol), alkaloids (e.g., nuciferine), and marine compounds [60,107,108]. Some flavonoids have been shown to inhibit P-gp via different mechanisms, including a decrease in P-gp expression [109], interference with ATP hydrolysis [110], and blockade of the binding site [111], thereby inhibiting the efflux of chemotherapeutic drugs from cancer cells and reversing their chemoresistance. For example, quercetin has been shown to sensitize gastric cancer cells to daunorubicin by downregulating the *ABCB1* gene, thereby reducing its overexpression and inhibiting its efflux from cancer cells, while enhancing their apoptosis [112]. Additionally, quercetin may affect the function of P-gp by disrupting signal transduction from the nucleotide-binding domain (NBD) to the transmembrane domain (TMD) [113]. Quercetin also downregulates P-gp expression in doxorubicin-resistant breast cancer cells, thereby enhancing the anti-tumor activity of specific chemotherapeutic agents, including doxorubicin, paclitaxel, and vincristine. Moreover, the number of breast cancer stem cells (BCSCs) significantly decreased during combined treatment, thereby indicating that this natural compound can reverse the MDR phenotype of cancer cells through regulating P-gp expression and eliminating BCSCs mediated by YB-1 nuclear translocation [114]. Besides ABCB1, quercetin also inhibited the activity of other types of ABC transporters, including ABCC1 and ABCG2 [115,116,117,118]. Chalcones, known as the precursors for the synthesis of flavonoids, are also shown to reverse MDR phenotype in cancer cells by modulating the expression of P-gp [119]. As a result, chalcones effectively potentiated the tumor-inhibitory effect of 5-fluoracil (5-FU) in a 5-FU-resistant human hepatocellular tumor xenograft model [120]. Quinine also reversed doxorubicin resistance in myeloma cells by inhibiting P-gp-mediated efflux [121]. Similarly, indole alkaloids such as indole-3-carbinol and indole-3-carbaldehyde effectively inhibited P-gp-mediated efflux of several chemotherapeutic drugs, including doxorubicin and vincristine, from cancer cells [122]. The detailed analysis of the P-gp-reversing potencies of natural compounds is beyond the scope of this review and has been addressed in detail in several fundamental reviews [60,107].

## 8. MRP1 (ABCC1) Inhibitors

Due to the well-documented impact of MRP1 overexpression on cancer development and drug resistance [123], a significant effort has been made to develop potent and selective MRP inhibitors for clinical oncology applications [124,125,126,127].

Although several MRP1 inhibitors have been developed over the past few decades, they are rarely specific to this target. MK-571 is the most commonly used inhibitor of multidrug resistance protein 1 (MRP1/ABCC1), which was initially developed as a cysteinyl leukotriene receptor 1 (CysLTR1) antagonist to treat patients with bronchial asthma [128]. Further studies demonstrated MK-571’s ability to increase the intracellular accumulation of MRP1 substrates [129], which may be a way to overcome the chemoresistance of cancer cells overexpressing ABCC1, but not ABCB1 [130]. Indeed, MK-571-induced inhibition of MRP1 resulted in a significant increase in vincristine- and etoposide-induced cell death in glioblastoma multiforme (GBM) cell lines in vitro, thereby providing a rationale for improving currently used chemotherapeutics for the initial treatment of primary and recurrent GBM [131,132,133]. In contrast, specific MRP1 knockdown or treatment with MK-571 did not affect the temozolomide-induced response in GBM cells, which was in concordance with previous findings, illustrating that temozolomide is a substrate for P-gp and Breast Cancer Resistance Protein (BCRP) but not MRP1 [134]. Interestingly, inhibition of cyclic nucleotide-specific phosphodiesterases, which are frequently upregulated in GBM and thus considered a promising avenue for GBM therapy, was significantly potentiated by MRP1 inhibition, thereby providing a new combination therapy for this malignancy [135]. Although subsequent studies have shown that MK-571 also modulates transport by other ABC transporters, including ABCB1 [136], thereby illustrating a lack of selectivity, MK-571 remains the most widely used small-molecule inhibitor of MRP1.

## 9. BCRP/ABCG2 Inhibitors

The first ABCG2 inhibitor reported was Fumitremorgin C (FTC), a mycotoxin derived from *Aspergillus fumigatus* [137,138]. Despite the potent anti-cancer activity of FTC in vitro, due to its ability to increase the intracellular accumulation of chemotherapeutic agents, the in vivo use of FTC was precluded because of its neurotoxicity. Thus, FTC analogs have been developed to decrease their toxic effects while retaining ABCG2-inhibitory activity [139]. This list includes Ko-143, which exhibited potent activity against multiple cancer cells by inhibiting the efflux of certain chemotherapeutic drugs, including mitoxantrone, etoposide, and paclitaxel via ABCG2 inhibition. Notably, Ko-143 was shown to be a non-selective ABCG2 inhibitor, inhibiting this ABC transporter more strongly than ABCB1 and ABCC1 [140,141]. To date, a long list of ABCG2 inhibitors has been identified [70,142,143]. Nevertheless, the clinical use of most of the ABCG2 inhibitors has not yet been achieved due to concerns about their safety and/or in vivo efficacy, thereby limiting their clinical development.

Thus, the use of drugs developed for other purposes and already existing on the market may be an attractive option for inhibiting the function of ABC transporters and thereby overcoming cancer resistance to chemotherapies. This might be the drugs known as substrates for ABC transporters and hence serving as their competitive inhibitors. In particular, this may include tyrosine kinase inhibitors (TKIs) designed to specifically target and inhibit the overactivated pathways in cancer cells, thereby leading to uncontrolled proliferation and enhanced survival [144,145]. Therefore, TKIs repurposing into cancer therapies and using their “off-target” effects may open up further avenues for combination therapies aiming to overcome cancer chemoresistance and improve OS and PFS rates of cancer patients, including those with resistant forms of malignant neoplasms [144,145].

## 10. “Off-Target” Effects of Tyrosine Kinase Inhibitors (TKIs) and Cancer Resistance to Chemotherapy

Besides the approaches targeting the aforementioned regulators of cancer chemoresistance, of specific interest are the inhibitors of receptor and non-receptor tyrosine kinases (TKIs) that were initially designed to inhibit particular signaling pathways and cascades activated in the neoplasms. Indeed, TKs facilitate essential biological cellular events, including proliferation, cell cycle progression, differentiation, migration, and metabolism. Conversely, changes in TK-mediated pathways, such as autocrine or paracrine stimulation, activating mutations, and hyperexpression, may lead to malignant transformation, progression, metastasis, and the development of resistance to chemotherapies [146,147]. In this vein, a wide range of TKIs is being developed and studied in clinical trials, and eventually, they were successfully introduced into the clinic, leading to significant improvements in clinical outcomes for patients with diverse types of malignancies [148]. On the other hand, the accumulating body of evidence suggests that the “off-target” effects of TKIs may be potentially utilized to overcome chemoresistance in malignancies by acting on other aspects of cancer cell behavior beyond the activation of TK-mediated pathways. In particular, several TKIs exhibiting their activities against different kinase types have also been shown to impair DNA damage repair (DDR) pathways, inhibit the pro-survival signaling, target cancer stem cells (CSCs), normalize the tumor microenvironment (TME), and inhibit efflux pump function by acting directly on ABC transporters [144,145]. As described above, TKIs can act as both substrates and inhibitors of ABC transporters, depending on their concentration and affinity to the transporter. Typically, TKIs at lower concentrations exhibit substrate-like properties. In contrast, at higher concentrations, they inhibit the function of the transporter, thereby increasing the concentration of substrate anti-cancer agents in vitro and in vivo. A detailed analysis of each group of TKIs that potentially affects the aforementioned mechanisms and resensitizes cancer cells to conventional chemotherapies is beyond the scope of this review. In this review, we will describe in detail the “off-target” activities of TKIs targeting fibroblast growth factor receptors (FGFRs) and vascular endothelial growth factor receptors (VEGFRs). As will be shown below, for each of the TKIs listed below, specific mechanisms have been identified that increase the sensitivity of cancer to chemotherapeutic agents, thereby opening novel therapeutic windows for cancer therapy.

## 11. Fibroblast Growth Factor Receptor (FGFR) Inhibitors

The FGF/FGFR signaling pathway is involved in embryonic development, angiogenesis, tissue homeostasis, and wound healing, while playing a regulatory role in cellular differentiation, proliferation, apoptosis, and migration. However, the abnormalities of FGF/FGFR signaling can also promote a broad spectrum of human diseases, including malignancies, predominantly due to the *FGFR* gene amplifications, mutations, and rearrangements/fusions [149,150,151]. This has led to the development of multiple therapeutic strategies targeting the FGF/FGFR pathway, which includes small molecular FGFR inhibitors (FGFRIs) and/or neutralizing antibodies. Several selective FGFR inhibitors have completed clinical trials and been approved for the treatment of human malignancies that exhibit *FGFR* abnormalities. In particular, pemigatinib and futibatinib were approved for the treatment of unresectable, previously treated, locally advanced, or metastatic cholangiocarcinoma (CCA) with *FGFR2* fusion [152,153]. Similarly, infigratinib has demonstrated efficacy for the treatment of advanced and metastatic CCA [154,155]. Additionally, pemigatinib was also approved for the therapy of relapsed or refractory myeloid/lymphoid neoplasms (MLNs) with *FGFR1* rearrangement [156]. Finally, erdafitinib is currently approved for the treatment of locally advanced or metastatic urothelial carcinoma (UC) [157].

**Figure 2:**
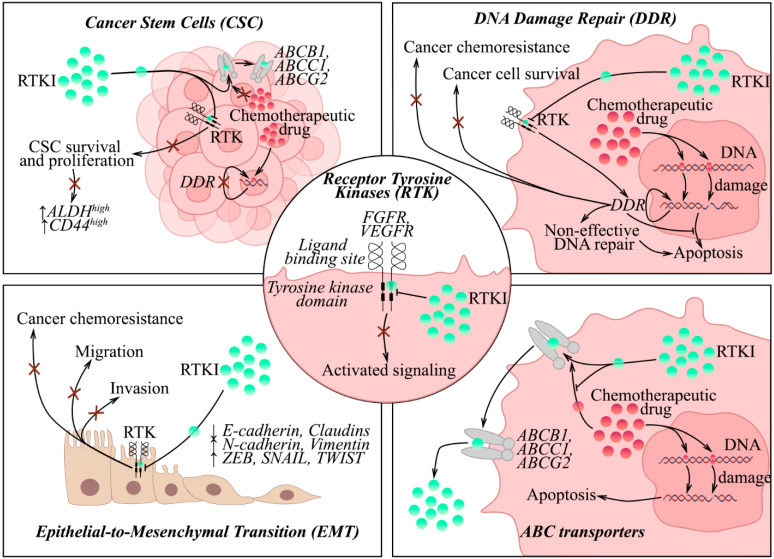
Targeted and non-targeted (i.e., “off-target”) effects of receptor tyrosine kinase inhibitors (RTKIs) modulating cancer chemosensitivity. ✕: inhibition of the corresponding pathways and activities.

Besides targeting the aberrantly activated FGF/FGFR pathway in the malignancies mentioned above, FGFRIs exhibited potent activities against diverse signaling pathways involved in cancer chemoresistance and beyond the FGFR pathway. This is due to the ability of FGFR-signaling to regulate DNA damage repair (DDR) cascades, EMT, and maintain the pool of cancer stem cells (CSCs). Additionally, some FGFRIs can serve as competitive inhibitors of ABC transporters (Figure 2), thereby making them an attractive tool for overcoming cancer chemoresistance, as discussed below.

## 12. FGFR Inhibitors and DNA Damage Repair

Multiple reports, including our own, demonstrate the tight functional crosstalk between the activities of FGFR signaling and diverse DDR pathways. In particular, pharmacological inhibition of FGFR signaling in cisplatin-resistant ovarian cancer cell lines and primary cell lines from patients with drug-resistant ovarian cancer effectively reversed drug resistance by attenuating high-fidelity homology-mediated DNA damage repair [158]. Mechanistically, FGF1 accelerates homology-mediated DNA repair by promoting the focal recruitment of replication protein A (RPA) to sites of single-stranded DNA (ssDNA) and stimulating the phosphorylation of ATM kinase, a well-known DNA damage sensor [158].

In concordance with these findings, we observed earlier that inhibition of FGFR2 signaling effectively attenuates homology-mediated DNA repair in cancer cells and sensitizes them to DNA-damaging agents (e.g., DNA topoisomerase II inhibitor doxorubicin) [159]. Of note, FGFR inhibition/depletion did not reduce the number of BrdU and phospho-RPA foci after doxorubicin treatment, suggesting that inhibition of FGFR signaling has no impact on the processing of double-strand breaks (DSBs) [159]. In contrast, the number of doxorubicin-induced Rad51 foci was decreased when FGFR2-mediated signaling was interrupted/inhibited by *siRNA FGFR2* or infigratinib, a pan-FGFR inhibitor. Moreover, Rad51 and γ-H2AX foci were mislocalized in FGFR-inhibited GIST, and the amount of Rad51 was substantially decreased in H2AX-immunoprecipitated complexes, thereby illustrating the defect of Rad51 recombinase loading to the doxorubicin-induced DSBs [159].

FGFR-mediated maintenance of effective homology-mediated DNA repair has also been recently demonstrated in breast cancer, as well as its resistance to poly (ADP-ribose) polymerase (PARP) inhibitors (PARPi). In particular, FGFR3 phosphorylates PARP1 at tyrosine 158, which is correlated with PARPi resistance in patient-derived xenograft models of triple-negative breast cancer (TNBC) and primary tumors. Mechanistically, phosphorylation of PARP1 at Tyr 158 facilitates BRG1-mediated HR by prolonging the loading of MRE11 and thereby promoting the resistance to PARPi. As a result, FGFR inhibition prolongs PARP trapping and synergizes with PARPi in breast cancer [160].

Additionally, FGFR inhibitors (e.g., AZD4547) were found to block DNA repair coordinated by PTEN, thereby potentiating the radiosensitivity of glioblastoma. Mechanistically, FGFR inhibition prevented the phosphorylation of PTEN, and consequently, its interaction with chromatin and subsequent DNA repair function [161]. Similarly, pemigatinib, an FGFR1-3 inhibitor, effectively sensitized glioblastoma to radiation-induced DNA damage due to decreased irradiation-mediated DDR [162]. In contrast, erdafitinib, a selective and potent pan-FGFR1-4 tyrosine kinase inhibitor, effectively suppressed the TNBC tumorigenicity of triple-negative breast cancer cells by generating reactive oxygen species (ROS), inducing DNA damage, and ultimately triggering cell death [163].

Overall, this data illustrates the FGFR signaling pathway as a potential molecular target for reversing chemosensitivity in diverse types of human malignancies and highlights novel combination chemotherapy approaches for future clinical evaluation.

## 13. FGFR Inhibitors and ABC Transporters

**Erdafitinib** is a selective pan-FGFR tyrosine kinase inhibitor. On 19 January 2020, the Food and Drug Administration (FDA) approved this inhibitor for the treatment of locally advanced or metastatic urothelial carcinoma (mUC) with susceptible *FGFR3* or *FGFR2* alterations who have progressed after platinum-containing therapy (NCT03390504) [164].

Besides the high potency of erdafitinib against mUC, several recent studies have demonstrated its “off-target” effect in sensitizing cancer cells to certain chemotherapies, due to its ability to interact with ABCB1 and impair its function. Significant decreases in IC50 values of various chemotherapeutic drugs (paclitaxel, vincristine, cisplatin) and enhancements of their intracellular accumulation when used in combination with these TKIs were shown for a broad spectrum of ABCB1-overexpressing cancer cell lines [165,166]. The ability of erdafitinib to increase the intracellular concentration of aforementioned chemotherapeutic agents in cancer cells effectively resensitized them to chemotherapies and induced apoptotic cell death. Notably, erdafitinib did not alter the expression of this ABC transporter in cancer cells, suggesting that it inhibits the function of this transporter [165]. Indeed, erdafitinib was also found to stimulate ATPase activity of ABCB1 and interact with this protein at a similar position of the drug-binding site according to molecular docking results [165,166]. Notably, the ability of erdafitinib to sensitize cancer cells to chemotherapies was solely dependent on ABCB1 since this TKI has no significant effect on ABCG2-mediated resistance to topotecan or SN-38, known substrates for ABCG2 [165].

**Infigratinib** (BGJ398) is a selective pan-FGFR inhibitor that was approved by the FDA on 29 May 2021 for the treatment of patients with previously treated, unresectable locally advanced, or metastatic CCA with fibroblast growth factor receptor 2 (*FGFR2*) fusion or other rearrangement as detected by an FDA-approved test. The efficacy of infigratinib was demonstrated in a multicenter, open-label, single-arm trial that enrolled 108 patients who met the specified criteria. We reported for the first time that infigratinib also exhibited the off-target effect by enhancing the intracellular accumulation of several chemotherapeutic agents, including doxorubicin and paclitaxel. This effect was specific to ABCB1-overexpressing chemoresistant cancer cells. As an outcome, infigratinib effectively resensitized cancer lines to doxorubicin and paclitaxel, decreasing their viability and inducing apoptotic cell death. Moreover, cancer cells treated with paclitaxel and infigratinib simultaneously accumulated in M-phase, thereby illustrating that this RTKI potentiates paclitaxel’s ability to dysregulate cell cycle progression. The molecular mechanism of this phenomenon was due to the ability of infigratinib to bind with the drug-binding pocket (DBP) of the ABCB1 transporter, thereby attenuating the efflux of chemotherapeutic agents from cancer cells [167].

**Pemigatinib** is a selective inhibitor of FGFR1-4. In April 2022, the US Food and Drug Administration (FDA) granted accelerated approval of this TKI for previously treated patients with advanced or metastatic cholangiocarcinoma (CCA) harboring *FGFR2*) fusions and other rearrangements based on results from the phase II FIGHT-202 trial (NCT02924376) [168]. Additionally, this TKI completed phase II clinical trials for the treatment of patients with non-muscle-invasive bladder cancer, including those with recurrent low- or intermediate-risk tumors (NCT03914794), gastrointestinal cancer (NCT05651674), and pancreatic cancer (NCT03914794). Besides the high potency of erdafitinib against malignancies harboring *FGFR2* mutations and other rearrangements, several recent studies have demonstrated its “off-target” effect to sensitize cancer cells to certain chemotherapies due to its ability to interact with ABCB1 and impair its function.

Besides the targeted FGFR-inhibitory effect, this RTKI also demonstrated promising preclinical results when it was used in combination with conventional chemotherapies, including doxorubicin and paclitaxel. Indeed, this RTKI effectively sensitized several ABCB1-overexpressing cancer cells to the aforementioned chemotherapeutic agents, increasing their intracellular accumulation. This was shown for HELA-C2 cervical carcinoma, SW620/Ad300 colon adenocarcinoma cell lines, and ABCB1-overexpressing HEK293 human embryonic kidney cells. Despite pemigatinib’s failure to reduce ABCB1 expression in cancer cells and its lack of effect on ABCB1 subcellular localization, the molecular docking data demonstrated pemigatinib’s capacity to bind to the substrate-binding site of ABCB1, thereby illustrating the potential molecular mechanism by which anti-cancer drugs are retained in cancer cells [169].

## 14. FGFR Inhibitors and Epithelial-to-Mesenchymal Transition (EMT)

Multiple reports demonstrate the tight functional crosstalk between the FGFR pathway and EMT observed for a broad spectrum of human malignancies, thereby providing a rationale for targeting the FGFR pathway to overcome cancer chemoresistance. The regulatory role of the FGFR pathway in maintaining the EMT phenotype in cancer cells can be achieved via diverse molecular mechanisms. For example, FGFR1 was overexpressed in bladder cancer and promoted EMT in several urothelial carcinoma (UC) cell lines [170]. This can be attributed to the activation of MAPK and PLCγ. Additionally, COX-2 was transcriptionally upregulated in FGFR1-overexpressing cells, and this was associated with increased intracellular levels of prostaglandin E2, which, in turn, promoted migration. This allowed the authors to conclude that FGFR1 can regulate EMT in UC cells via the MAPK/PLCγ/COX-2 axis [170]. In concordance with these findings, FGF-Erk1/2 signaling in lapatinib-resistant cancer cells was shown to stabilize the EMT transcription factor Twist, thereby maintaining the EMT phenotype and mediating drug resistance. Importantly, cancer resistance to Her-2 inhibitors was successfully overcome by supplementing them with FGFR inhibitors, thereby providing a rationale for dual inhibition of these pathways [171]. Additionally, FGF4, a well-known ligand of FGFR2, has been shown to stimulate EMT during cancer progression by increasing store-operated calcium entry (SOCE) and the expression of the calcium signal-associated protein Orai1. As an outcome, Orai1 knockdown and the SOCE inhibitor 2,5-di-tert-butylhydroquinone (BHQ) effectively reversed all of the EMT-promoting effects of FGF4. The last one was also demonstrated in vivo on xenograft models [172]. Additionally, FGFR inhibitors, particularly PD173074, a well-known FGFR inhibitor, have also been shown to reverse the EMT phenotype of head and neck squamous cell carcinoma (HNSCC) cells and induce a mesenchymal-to-epithelial transition (MET) via the transcription factor AP-1, thereby suppressing cancer cell growth and invasion [173].

Overall, these studies demonstrate the FGFR pathway’s ability to maintain the EMT phenotype and, therefore, are implicated in cancer drug resistance beyond the areas of FGFR signaling. Indeed, FGFR inhibition in drug-tolerant EGFR-mutant non-small cell lung cancer (NSCLC) cells, harboring *EGFR T790 M*, effectively synergized with EGFR inhibitors and overcame EMT-mediated acquired drug resistance, thereby providing a rationale for combined FGFR and EGFR inhibition for EGFR-mutated cancer cells [174]. Similarly, cancer resistance to Her-2 inhibitors was successfully overcome when they were used in combination with FGFR inhibitors, thereby providing a rationale for dual inhibition of these pathways to overcome the resistance to Her-2 inhibitors [171].

Collectively, these studies clearly illustrate the high potency of the FGFR pathway in regulating EMT in diverse cancer cells and maintaining their aggressive phenotype, including resistance to chemotherapies. This, in turn, opens the novel therapeutic window of using FGFR inhibitors to overcome cancer chemoresistance via FGFR-mediated regulation of EMT.

## 15. FGFR Inhibitors and Cancer Stem Cells (CSCs)

Recent reports have shown that FGFR signaling may also be involved in regulating cancer stem cells (CSCs) and promoting cancer cell stemness, thereby making tumors resistant to chemotherapy agents. For example, FGF-2 was enriched in cisplatin-resistant head and neck CSCs (HNCSC) cells, which highly express the CD44 surface marker and exhibit high intracellular activity of aldehyde dehydrogenase (ALDH). As expected, infigratinib, a pan-FGFR small-molecule inhibitor, depleted the number of ALDH^high^CD44^high^ cells, thereby revealing a crucial role of FGFR signaling in maintaining the CSCs population in this particular type of cancer [175]. Similar activity was observed for AZD 4547, which effectively repressed ALDH activity and oncosphere formation in non-small cell lung cancer (NSCLC) cells both in vitro and in vivo [176]

Similarly, inhibition of FGFR signaling with infigratinib or dovitinib significantly decreased cell survival and proliferation of prostate cancer stem cells (CSCs) expressing stemness markers, including ALDH7A1 and OCT4 [177], and thereby suggesting the FGFR pathway as a promising therapeutic target in conjunction with current androgen deprivation therapy. In concordance with these findings, the role of the FGFR pathway, particularly FGFR1, is implicated in cancer cell stemness in luminal A breast cancer cells, promoting palbociclib resistance through the enhancement of Akt/Erk-ER signaling [178]. Similar findings were observed for esophageal squamous cell carcinoma (ESCC), highlighting the crucial role of FGF-2-mediated activation of the FGF/Erk signaling pathway in maintaining the CSCs. Notably, the Mek inhibitor, trametinib, effectively inhibited the CSCs. Thus, these findings also illustrate the essential role of FGF-2 in regulating CSCs via the Mek/Erk signaling pathway, thereby suggesting that inhibition of FGFR and/or Mek signaling represents a potential novel therapeutic option for targeting CSCs in ESCC [179]. Lastly, pemigatinib was also shown to decrease CSC survival and proliferation [162].

Overall, the studies mentioned above illustrate the crucial role of the FGFR pathway, particularly the overexpression of FGFR1, in maintaining CSCs in various types of human malignancies and thereby decreasing the effectiveness of conventional chemotherapies. This, in turn, opens up a novel therapeutic strategy to improve the clinical outcomes of cancer patients by combining certain chemotherapies with TKIs targeting the FGFR pathway.

## 16. Non-Selective FGFR Inhibitors

Besides selective FGFR inhibitors indicated above, the non-selective FGFRIs also target other receptor- and non-receptor TKs. In general, the non-selective FGFRIs possess their inhibitory activities against vascular endothelial growth factor receptors (VEGFRs) and platelet-derived growth factor receptors (PDGFRs). Obviously, dual inhibition of the PDGFR and VEGFR signaling pathways has the potential benefit of simultaneously targeting tumor cell proliferation and angiogenesis. Of note, the non-selective FGFRIs are generally less potent against the FGFR pathway, which limits their effectiveness regarding this specific target. Moreover, several studies, including our own, demonstrated a significant crosstalk between the FGFR and VEGFR pathways, thereby making this story more complex [180]. Similar to selective FGFRIs, several non-selective FGFRIs possess “off-target” activities beyond targeting of specific signaling pathways aimed as a primary molecular target (s) and effectively sensitize cancer cells to conventional chemotherapy. Dovitinib, ponatinib, lucitanib, lenvatinib, nintedanib, pazopanib, brivanib, regorafenib, cediranib, and intedanib are the most well-known multi-kinase inhibitors that target FGFR and exhibit activity against various RTKs beyond FGFRs. Several of them also exhibit inhibitory activities against ABC transporters, CSCs, EMT, and DDR, thereby increasing the chemosensitivity of cancer cells and overcoming cancer chemoresistance.

**Ponatinib** is a third-generation tyrosine kinase inhibitor (TKI) approved by the FDA to treat patients with relapsed or refractory Philadelphia chromosome-positive acute lymphoblastic leukemia (ALL) and chronic myeloid leukemia (CML), and the only approved inhibitor to successfully target BCR-ABL kinase T315I mutation in CML [181]. Moreover, ponatinib is a non-selective TKI that also targets FGFR1, VEGFR2, PDGFRα, Abl, RET, and Src kinases. Given that aberrant activation of FGFR1, VEGFR2, PDFGRα, Abl, and RET is related to other human malignancies, like lung and breast cancer, and medullary thyroid cancer, ponatinib-based targeted therapy may be beneficial for the aforementioned groups of cancer patients. Besides specific targeting of TKs indicated above, recent studies illustrated ponatinib’s potency to regulate activity and expression of several ABC transporters. Indeed, ponatinib is also a substrate of several ABC transporters, including P-gp and BCRP. This effect was observed in both in vitro and in vivo studies. In particular, mice lacking the expression of both P-gp and BCRP exhibited significantly higher brain-to-plasma ratios of ponatinib compared to wild-type animals, thereby highlighting the significant impact of ABC transporters on ponatinib’s biodistribution [182,183]. Further studies illustrated the role of *ABCB1* polymorphisms on ponatinib’s distribution into the cerebrospinal fluid in Philadelphia chromosome-positive ALL patients, thereby revealing ponatinib to be a substrate for ABCB1 [184]. Thus, ponatinib’s ability to interfere with the function of ABC transporters may be used to sensitize cancer cells to chemotherapies.

In concordance with these findings, ponatinib has been shown to increase the cytotoxicity of chemotherapeutic agents in BCRP- and P-gp-overexpressing cancer cells [185]. Similarly, ponatinib potentiated the cytotoxicity of anti-cancer drugs in MRP7-expressing cells by enhancing their intracellular concentrations. In particular, this was shown for microtubule-binding agents, paclitaxel, docetaxel, vincristine, and vinblastine, which are well-known substrates for MRP7. This was achieved by inhibiting MRP7 activity and downregulating MRP7 expression [186]. Besides the aforementioned activity of ponatinib against ABC transporters, this TKI also inhibited EMT, as evidenced by the upregulation of E-cadherin, a decrease in Vimentin and p-Smad3 [187], and an increase in NANOG and SOX2 [188]. Lastly, ponatinib was shown to be effective at eliminating leukemic stem cells in chronic myeloid leukemia (CML) patients, thereby suggesting that this TKI could be potentially helpful in achieving treatment-free remission in CML patients [189].

**Dovitinib** is a multi-kinase inhibitor targeting KIT, FGFR1-3, VEGFR1-3, and PDGFR A/B/, RET, and FLT-3 [190]. In contrast to ponatinib, dovitinib retained its efficacy in cell lines overexpressing P-gp or BCRP. Additionally, this TKI did not induce the expression of all major efflux transporters that confer multidrug resistance, thereby allowing the authors to propose that dovitinib might enhance co-administered anti-cancer drugs by suppressing MDR protein expression [191]. Indeed, further studies demonstrated that this TKI can potentiate the cytotoxic effects of DNA-damaging chemotherapies. For example, dovitinib synergizes with oxaliplatin in suppressing the proliferation of colorectal cancer (CRC) cells and inducing apoptosis, regardless of the RAS-RAF mutational status. Importantly, this combination also exhibited vigorous synergistic anti-proliferative activity and inhibition of angiogenesis in CRC xenograft models with MDR phenotype. Since the expression of ABC transporters and intracellular content of chemotherapeutic agents were not examined in this study, the authors proposed that simultaneous inhibition of the kinase-mediated signaling by dovitinib and oxaliplatin-induced DNA damage led to a significant decrease in angiogenesis and inhibited cell proliferation [192].

In consistency with these findings, further studies demonstrated dovitinib’s ability to impair DNA damage responses. In particular, dovitinib-treated glioblastoma (GB) primary cells and cell lines exhibited downregulation of key base excision repair (BER) factors and O6-methylguanine-DNA-methyltransferase (MGMT), which play crucial roles in repairing temozolomide (TMZ)-induced alkylating DNA damage. As a result, GB cells exhibited enhanced DNA damage, as assessed by nuclear γH2AX foci and comet assays, when treated with a combination of dovitinib and TMZ. Additionally, dovitinib reduced GB tumor sphere formation, potentially affecting cancer cell renewal and the number of CSCs in GB [193].

Dovitinib’s ability to sensitize cancer cells to DNA-damaging agents may also partly result from its capacity to target topoisomerase I and topoisomerase II, both of which are known as DNA processing enzymes [194].

Synergistic or additive effects of combining chemotherapy plus dovitinib were also observed in several preclinical models with patient-derived xenografts of breast, lung, and pancreatic cancer. In particular, a synergy was observed for combining dovitinib with paclitaxel or gemcitabine, but not with doxorubicin [195]. Interestingly, this inhibitor was also able to activate SNAI1/2-IFN-γ signaling axis and restrict the EMT program in gastric cancer, thereby converting immune desert-type tumors to immune-inflamed-type tumors, and sensitizing them to CTLA4 blockade [196].

**Pazopanib** is an orally active, second-generation TKI that targets c-KIT, FGFR, PDGFR, and VEGFR. It was approved by the United States Food and Drug Administration (FDA) for the treatment of metastatic renal cell carcinoma in October 2009. In April 2012, it was also approved for the treatment of advanced soft tissue sarcoma (STS), except for adipocytic STS and gastrointestinal stromal tumors (GISTs).

Similar to the RTKIs above, pazopanib was initially shown as a substrate for BCRP and, to a lesser extent, for P-gp. This was evidenced in vitro by using kidney cells overexpressing BCRP or P-gp and treated with specific BCRP and P-gp inhibitors (zosuquidar and Ko-143, respectively) [197]. Further studies demonstrated that pazopanib exhibits potent P-gp and BCRP-inhibitory activities, leading to the intracellular retention of other TKIs, e.g., dasatinib, and illustrating that combinations of TKIs that could enhance intracellular concentrations of the targeted TKI, overcome MDR, and improve TKI efficacy [198]. This point of view was also applied to combination therapy, which combines pazopanib with chemotherapeutic agents known as substrates for MDR proteins. For example, a combination of pazopanib with microtubule inhibitors, such as paclitaxel, produced synergistic anti-tumor effects in anaplastic thyroid cancer (ATC) cells and xenografts, thereby illustrating a promising therapeutic approach in ATC [199]. Additionally, a single (pilot) ATC patient with lung metastases treated with this combination of chemotherapeutic drugs attained durable regression of metastatic disease (confirmed RECIST response).

In consistency with in vitro studies, several trials revealed the efficacy of these combinations. For example, the effectiveness of paclitaxel and pazopanib was assessed in patients with advanced solid tumors, demonstrating that 23% of patients had a partial response, and 58% achieved stable disease. Moreover, the combination of pazopanib and paclitaxel resulted in a 26% greater geometric mean paclitaxel area under the curve, suggesting synergistic activity and providing a further basis for a phase II clinical study [200]. Indeed, phase II of some trials illustrated that the combination of paclitaxel and pazopanib resulted in a promising overall response rate (ORR) of 54% in patients with relapsed or refractory urothelial carcinoma (UC) who progressed after two previous chemotherapeutic regimens [201]. A phase II single-arm, open-label clinical trial demonstrated the benefits of combining pazopanib with metronomic paclitaxel to enhance its antiangiogenic effects for patients with unresectable stage III and stage IV melanoma. The combination was well tolerated and resulted in a 37% objective response rate, an overall clinical benefit of 93%, a median of PFS of 8 months, and OS of 12.7 months [202].

Nevertheless, the advantages of combining conventional therapies with pazopanib were not evidenced in other clinical trials, which did not show significant clinical benefits of its combination with chemotherapies compared to chemotherapies alone. This was shown for paclitaxel for therapies of angiosarcoma [203] and persistent or recurrent ovarian cancer [204].

Despite the initial promising results of the pilot study shown above, which illustrated the clinical benefit of pazopanib used in combination with paclitaxel for patients with APC, further studies did not confirm this finding. Indeed, the addition of pazopanib to paclitaxel and radiotherapy was feasible and safe, but it did not benefit patients with APC regarding the improvement of overall survival (OS) [205].

Besides its activity against the aforementioned TKs and ABC-transporters, pazopanib generates ROS-induced DNA damage in renal cell carcinoma (RCC), as evidenced by increased expression of γH2AX [206]. Moreover, this inhibitor promoted nuclear translocation of nuclear factor E2-related factor 2 (Nrf2), increased cellular senescence, and reduced telomerase activity, thereby illustrating a novel molecular mechanism utilized by pazopanib to display its cytotoxicity against RCC cells [206]. Lastly, pazopanib inhibited several VEGFa-induced activities in cervical cancer (CC) cells, including EMT, migration, invasion, and anoikis resistance in CC cells [207].

**PD173074** is a potent inhibitor of FGFR1 and VEGFR, and it was also shown to effectively resensitize chemoresistant cancer cells overexpressing ABCB1 and ABCC10 to some chemotherapeutic agents, including paclitaxel. Despite the efflux of the specific substrates being decreased in PD173074-treated cancer cells, the expression of the aforementioned ABC transporters remained constant, thereby suggesting the ability of this TKI to impair the function of P-gp and MRP7 [208,209]. This group of scientists also further demonstrated that PD173074 reversed the MRP7-mediated MDR phenotype in MRP7-overexpressing cells, inhibiting the efflux of paclitaxel, a well-known substrate for MRP7 [209]. Similarly, PD173074 potentiated the anti-tumor activity of 5-fluorouracil in gastric cancer cells by reducing their proliferation and inducing apoptosis [210]. Notably, PD173074 stimulated the ATPase activity of ABCB1 in a concentration-dependent manner, thereby demonstrating its direct interaction with the ABCB1 transporter. Interestingly, PD173074 failed to inhibit photolabeling of ABCB1 protein with [125I]-iodoarylazidoprazosin (IAAP), thereby suggesting that PD173074 has a different site from that of IAAP in the drug-binding pocket [208]. Similarly, PD173074 effectively potentiated the cytotoxic activities of paclitaxel and doxorubicin in endometrial cancer cell lines. Notably, this was not achieved due to inhibition of the FGFR signaling pathway, as the cancer cells used in this study exhibited wild-type FGFRs and were therefore resistant to FGFR inhibition alone [211].

In addition to its inhibitory activity on ABC transporters, PD173074 has also been shown to induce the mesenchymal-to-epithelial transition (MET). This was observed in head and neck squamous cell carcinoma (HNSCC) cells treated with PD173074. Mechanistically, this TKI suppressed ERK1/2 and p38 activation, thereby inhibiting phosphorylation of c-Jun and decreasing expression of Snail1. Moreover, reduced expression of Snail might be due to PD173074-induced stimulation of GSK3β activity, which inhibits transcription of Snail1 and increases E-cadherin expression [173].

Besides those mentioned above, “off-target” effects of PD173074 in potentiating the cytotoxic effects of DNA-damaging therapy, this FGFR inhibitor has also been shown to be a potent antagonist of high mobility group-AT hook 2 (HMGA2). This non-histone architectural transcription factor is highly overexpressed in various types of cancer, thereby promoting cell cycle progression and proliferation. Thus, binding of PD173074 to the C-terminal domain of HMGA2 modulates the DNA-binding mode of HMGA2 and interferes with HMGA2–protein interactions [212].

## 17. Vascular Endothelial Growth Factor Receptor (VEGFR) Inhibitors

Vascular endothelial growth factors (VEGFs) and their receptors (VEGFRs) regulate angiogenesis and vascular remodeling, thereby playing a crucial role in the regulation of diverse physiological and pathological conditions. In particular, VEGFR signaling effectively regulates endothelial cell (EC) proliferation, migration, and survival, playing an essential role in new vessel formation, which is critical for embryogenesis, wound healing, inflammation, metabolic and reproductive disorders, ocular and cardiovascular diseases, and carcinogenesis, as well [213,214].

In particular, angiogenesis is essential for solid tumor growth and metastasis and can be regulated by a balance of pro- and antiangiogenic factors. Therefore, to promote angiogenesis, tumor and stromal cells produce and secrete VEGFs, thereby activating VEGFR1 and VEGFR2 on tumor EC [215]. Additionally, several oncogenes, including RAF, RAS, and mutated p53, were shown to regulate VEGF expression [216,217]. This made angiogenesis an attractive therapeutic target and boosted the development of small-molecule inhibitors and/or neutralizing antibodies targeting this overactivated pathway in multiple malignancies. Notably, besides inhibiting tumor vasculature growth, VEGFR inhibitors have a direct anti-tumor effect, interfering with cancer cell survival [218], and can also induce vessel normalization [219], thereby improving the blood flow and providing effective delivery of anti-cancer agents to solid tumors. Additionally, anti-VEGF therapy modulates the functions of a broad spectrum of immune cells, including T-lymphocytes and dendritic cells, thereby orchestrating the development of appropriate anti-tumor immunity [220,221]. Lastly, antiangiogenic therapy can sensitize cancer cells to chemotherapy through a broad spectrum of molecular mechanisms, which will be discussed in detail in a specific subchapter indicated below.

A broad spectrum of VEGF/VEGFR inhibitors has completed clinical trials and are currently approved for the treatment of human malignancies. This includes anti-VEGF-A monoclonal antibodies (mAbs) (e.g., bevacizumab) and VEGFR2 (e.g., ramucirumab). The first one is mAbs binding to VEGF-A, preventing it from activating VEGF receptors on the surface of endothelial cells. Bevacizumab was the first FDA-approved VEGF inhibitor, which is currently approved for therapy of a broad spectrum of human malignancies, including CRC (2004), NSCLC (2006), metastatic breast cancer (2008), GB (2009), renal cell carcinoma (2009), cervical cancer (2014), and ovarian cancer (2018) [222,223,224]. The second one is an anti-VEGFR2 antibody, blocking VEGF ligands from activating the VEGFRs, thereby inhibiting angiogenesis, and it is approved for therapy of gastric cancer (2014), NSCLC, including EGFR-mutated and metastatic forms (2014, 2020), CRC (2015), and HCC (2019) [225,226].

The list of small-molecule VEGFR inhibitors currently approved for cancer therapy is much broader. It includes pazopanib, sunitinib, sorafenib, axitinib, cabozantinib, regorafenib, lenvatinib, apatinib, ponatinib, and vandetanib, among others. Some of them (e.g., axitinib) are known as selective and therefore potent VEGFR inhibitors. Despite their high potency in inhibiting the VEGFR pathway, the vast majority of them are non-selective TKIs, also exhibiting potent inhibitory activities against a broad spectrum of RTKs and non-RTKs. For example, almost all of them inhibit KIT and PDGFRα/β signaling, whereas inhibition of the FGFR pathway has been proven for ponatinib, lenvatinib, regorafenib, and nintedanib. Several of them also effectively target some of the non-RTKs, including RET and FLT-3 (e.g., cabozantinib, sunitinib, regorafenib, and sorafenib), thereby broadening the range of human malignancies suitable for this targeted therapy. In particular, this includes renal cell carcinoma, soft tissue sarcomas, gastrointestinal stromal tumors, hepatocellular carcinoma, colorectal cancer, etc [227,228,229,230,231,232].

As was shown for FGFR inhibitors, non-selective VEGFR inhibitors also exhibit the “off-target” activities beyond the VEGF/VEGFR signaling pathway and potentiate the cytotoxic effects of chemotherapeutic agents. These activities underlie the regulation of EMT and diverse DDR pathways, the inhibition of ABC transporter activities, and the maintenance of the pool of CSC.

Indeed, **axitinib**, a potent tyrosine kinase inhibitor of VEGF receptors, approved for advanced renal cell carcinoma (RCC), was shown as a substrate for BCRP and MDR1 in in vivo and in vitro analyses [233]. Following studies confirmed that adverse events leading to discontinuation or dose reduction in axitinib were associated with increased axitinib plasma exposure; however, this was not directly related to genetic polymorphisms of ABC transporters [234]. In consistency with these findings, axitinib reversed *ABCG2*-mediated MDR in cancer cells in vitro by enhancing the cytotoxicity of topotecan and mitoxantrone in human lung cancer cell lines. Importantly, this happened without altering the expression of *ABCG2* at the mRNA or protein levels. In agreement with in vitro data, axitinib significantly potentiated the anti-tumor activity of topotecan in nude mice bearing *ABCG2*-overexpressing tumor xenografts. It was also proposed that this effect was due to axitinib’s ability to target cancer stem-like cells and thereby enhance the efficacy of chemotherapeutic agents [235]. Of note, when used at higher concentrations, axitinib also exhibits inhibitory activities against c-KIT and PDGFRβ, thereby raising a concern about its selectivity against the VEGFR pathway. Additionally, axitinib exhibited both cytotoxic and immunomodulatory effects on RCC cells, contributing to its anti-tumor activity [236,237]. In particular, axitinib treatment of RCC cells induced a DNA damage response (DDR) characterized by γ-H2AX phosphorylation and Chk1 kinase activation, which was followed by p21 activation. This resulted in G2/M/M arrest and development of a senescent-like phenotype accompanied by enlargement of cells and increased senescence-associated activity of β-galactosidase [236,237]. Besides this, axitinib also inhibited EMT in breast cancer cells [238] and targeted CSC in RCC [239].

Similarly, lenvatinib and motesanib exhibit potent VEGFR inhibitory activity. However, they also exhibit cross-inhibitory activities against other RTKs and non-RTKs (e.g., PDGFRβ, FGFR1-4, RET, and c-Kit) at higher concentrations. Thus, the vast majority of VEGFR inhibitors are known as the non-selective RTKIs, targeting a broad spectrum of signaling cascades that regulate cell proliferation and survival. Given that several of the non-selective RTKIs exhibit inhibitory activities against both FGFR and VEGFR pathways (e.g., pazatinib, dovitinib, and regorafenib), their “off-target” effects increasing chemosensitivity of cancers were discussed above in the corresponding chapter. The “off-target” effects of other RTKIs are shown below, inhibiting VEGFR and other signaling pathways (i.e., without affecting the FGFR pathway).

**Regorafenib** is a sorafenib-derived multi-kinase inhibitor that targets tumor cells and their microenvironment due to inhibition of angiogenic (VEGFR1-2 and TIE-2), stromal (PDGFRβ and FGFR1), oncogenic (RET and c-KIT), and intracellular (BRAF, c-RAF/Raf-1) kinases. Regorafenib was approved as a second-line therapy for advanced hepatocellular carcinoma (HCC) [240] and as a third-line treatment for advanced colorectal cancer (CRC) [241,242] and gastrointestinal stromal tumors (GISTs) [243]. Multiple reports also indicate that this TKI exhibits activities beyond inhibiting the TKs above, which helps overcome cancer resistance to the chemotherapeutic agents.

On the one hand, this is rooted in the MDR-related area and is due to regorafenib’s ability to interact with ABC transporters, thereby impairing their function and decreasing the efflux of chemotherapeutic agents from cancer cells. For example, regorafenib significantly improved the sensitivity of BCRP-overexpressing S1-M1-80 CRC cells to mitoxantrone and SN-38, both substrates of BCRP, by interacting with the BCRP transmembrane domain and impairing BCRP efflux, thereby increasing intracellular drug retention [243]. Regorafenib also synergized with topotecan in vivo, exhibiting potent anti-tumor activity against BCRP-overexpressing CRC xenografts [244]. Similarly, regorafenib enhanced cell sensitivity to paclitaxel, a substrate of ABCB1, by increasing the intracellular paclitaxel level via inhibition of ABCB1-associated chemotherapy efflux. Lastly, regorafenib effectively overcame MDR in ABCB1-overexpressing CRC models both in vitro and in vivo [245]. Of note, several reports indicate that regorafenib can be a substrate for P-gp and BCRP, thereby suggesting its ability to sensitize cancer cells to certain chemotherapies acting as a competitive ABC inhibitor [246,247].

On the other hand, an accumulating body of evidence demonstrates regorafenib’s ability to attenuate DNA repair and thereby potentiate the effects of DNA-damaging therapies, including chemotherapy and radiotherapy. For example, regorafenib suppressed radiation-induced DNA damage repair in triple-negative breast cancer (TNBC) cells and enhanced their radiosensitivity in a time-dependent manner [248]. Regorafenib also attenuated DDR mechanisms and potentiated the efficacy of PARP inhibitors in pancreatic ductal adenocarcinoma with germline *BRCA1/2* mutations [249]. Interestingly, regorafenib, when used alone, induced DNA damage and reduced the capacity of PDAC cells to repair this damage. However, regorafenib alone did not induce apoptosis in PDAC cells. Strikingly, when regorafenib was used in combination with olaparib, a substantial increase in DNA damage was registered in vitro, in tumor-bearing mice, and in ex vivo PDAC cultures, resulting in increased apoptosis compared to olaparib alone [249]. Regorafenib’s ability to cause DNA damage in cancer cells was confirmed in subsequent studies, whereas the activation of DNA repair mechanisms promoted resistance to this TKI [250]. Regorafenib also promoted anti-tumor progression in melanoma by reducing ribonucleotide reductase subunit 2 (RRM2), which is required for DNA repair and replication [251].

Additionally, regorafenib regulates the EMT, but the data in this field are conflicting. On the one hand, regorafenib suppressed EMT in cholangiocarcinoma (CCA) cells by inhibiting the activity of yes-associated protein 1 (YAP1) and regulating EMT-related genes, including E-cadherin and SNAIL [252]. Opposing results were shown by Kehagias P. and co-authors, illustrating regorafenib inducing EMT and senescence in CRC cells. Significantly, these factors promoted resistance to this TKI [253]. Lastly, regorafenib impacts the stemness phenotypes in colorectal cancer (CRC) cells by targeting tumor sphere formation, the expression of stemness markers, and the side-population [254].

**Cabozantinib** is a multitargeted tyrosine kinase inhibitor that primarily affects the activity of VEGFR2 and MET. It is approved for the therapy of several solid tumors, including advanced renal cell carcinoma and medullary thyroid cancer [255,256]. Besides targeting these kinases, several reports also demonstrated the potency of enhancing the cytotoxic anti-cancer activities of several chemotherapeutic agents. This might be due to the targeting of ABC transporters. For example, cabozantinib potentiated the cytotoxic properties of topotecan, mitoxantrone, and SN-38 in mitoxantrone-resistant NSCLC subline and several ABCG2-overexpressing cells [257]. This was due to inhibition of ABCG2-mediated efflux of the aforementioned chemotherapeutic agents from cancer cells without affecting the expression of ABCG2 [257]. This was in concordance with recent findings illustrating that several MET inhibitors, including cabozantinib, crizotinib, and PHA665752, potentiated the cytotoxic effect of mitoxantrone in breast cancer cells via inhibiting the ABCG2-mediated efflux of this chemotherapeutic agent [258]. Similarly, cabozantinib potentiated the cytotoxic and anti-tumor effects of topotecan, a well-known substrate for ABCG2, in chemoresistant NSCLC cells in vitro and xenograft models, as well. This RTKI effectively reversed the sensitivity of drug-resistant ABCG2-expressing NSCLC cells to topotecan by restoring its intracellular accumulation via inhibiting ABCG2 function [259].

Of note, the ABC-inhibitory abilities of cabozantinib are not limited to the ABCG2 transporter. In particular, cabozantinib reversed resistance to doxorubicin, a substrate for P-gp, in human hepatoma cells both in vitro and in vivo on hepatoma xenografts in mice. This TKI effectively stimulated the activity of P-gp ATPase and did not change the expression of P-gp mRNA or protein. Lastly, cabozantinib and verapamil exhibited partial overlap in a binding site on P-gp, thereby supporting cabozantinib’s ability to interfere with the function of P-gp [260].

Several studies also illustrated cabozantinib’s impact on CSCs and EMT. In particular, cabozantinib-treated pancreatic ductal adenocarcinoma (PDA) cells exhibited downregulation in CD133, a well-known CSC marker, probably due to its downregulation of pluripotency transcription factor SOX2, as well as induced apoptosis of cancer cells treated with gemcitabine for an extended period of time [261]. Although the authors did not examine whether co-treatment of PDA cells with cabozantinib and gemcitabine may prevent the development of gemcitabine resistance, cabozantinib-mediated prevention of enrichment of CSCs during repeated gemcitabine treatment might be a promising therapeutic avenue to overcome PDA resistance to gemcitabine. Cabozantinib has also been demonstrated to inhibit EMT induced by TGF-β in breast cancer cells [262], thereby complementing the molecular mechanisms of cabozantinib-induced sensitization of chemoresistant human malignancies to certain chemotherapies.

**Sunitinib** is a multi-kinase inhibitor, targeting PDGFR, VEGFR, Kit, and FLT-3. This inhibitor was approved by the FDA for the treatment of renal cell carcinoma (RCC) and imatinib-resistant GIST in 2006. Besides its high potency to inhibit the corresponding TKIs, sunitinib was shown to reverse ABCG2-mediated resistance to topotecan and doxorubicin in vitro [263]. Additionally, sunitinib also inhibited ABCB1 function, but to a much lesser extent when compared with ABCG2 [264].

Interestingly, sunitinib was also shown to induce DNA damage, which was evidenced by increased expression of γ-H2AX, a well-known marker of double-strand breaks and formation of micronuclei. Moreover, this was associated with decreased expression of DNA repair proteins, including Rad51 recombinase, thereby providing the molecular basis for sunitinib-induced enhancement of cancer sensitivity to DNA-damaging therapies due to the attenuation of homology-mediated DNA damage repair mechanisms [265]. The others have also described the DNA-damaging properties of sunitinib. In particular, sunitinib-induced DNA damage in metastatic RCC cells led to cell cycle arrest and senescence and the development of senescence-associated secretory phenotype (SASP), thereby revealing the ability of this TKI to attenuate DDR repair capacities in cancer cells and suggesting the therapeutic significance of sunitinib-induced RCC cellular senescence [266]. Recent findings also revealed the DNA-damaging properties of sunitinib, which were associated with an increased extracellular ROS production, p53 activation, and downregulation of Bcl-2 [267]. In concordance with these findings illustrating sunitinib’s ability to enhance cancer cell sensitivity to DNA-damaging therapies, radiosensitization was shown for esophageal squamous cell carcinoma cells treated for 24 h with sunitinib before irradiation, thereby highlighting this RTKI as a promising agent for future clinical trials with chemoradiation in esophageal cancer [268].

Surprisingly, sunitinib was also shown to upregulate EMT in cancer cells, thereby illustrating that although VEGFR-targeting antiangiogenic agents play an essential role in the therapy of some human malignancies, they may promote disease progression by stimulation of invasion and metastasis of cancer cells. Indeed, sunitinib was shown to promote EMT in CRC cells and increase their motility and migration [269]. This is in concordance with our recent findings, which illustrated the similar effect of imatinib mesylate (IM) on IM-resistant GIST cells, and thereby suggesting that continuation of IM-based therapy for resistant GIST promotes disease progression [270]. Similarly, it was found that sunitinib could have adverse effects of promoting RCC progression by increasing vascular mimicry (VM) formation of RCC cells [271]. These results are in contrast with previous studies, generally illustrating the decreased motility and proliferation rate in sunitinib-treated cancer cells.

Similar to EMT, conflicting reports are currently available to illustrate sunitinib’s impact on CSC regulation. Initially, sunitinib was shown to deplete CSC and thereby enhance sensitivity to DNA-damaging therapies. For example, sunitinib effectively reduced the number of ALDH^high^ cancer stem-like cells and sensitized these cells to radiation-mediated loss of clonogenicity in prostate cancer models in vitro and in vivo [272]. This was consistent with other findings illustrating sunitinib’s ability to decrease CSC proliferation [239] and differentiation in vitro and in vivo [273]. All these findings are in contrast with recent data illustrating sunitinib-increased CSC-like phenotype in RCC cells via upregulation of lncRNA-ECVSR/ERβ/Hif2-α signaling [271]. Overall, this data illustrated the complexity of the molecular mechanisms underlying the sunitinib-based regulation of chemoresistance and disease progression.

Several other multi-kinase inhibitors exhibiting activity against the VEGFR pathway were also shown to be the targets and/or inhibitors of ABC transporters. This includes vandetanib [274,275], vatalanib [276], anlotinib [277], apatininb [236], motesanib [278], nintedanib [237], Telatinib (BAY 57-9352) [279], SKLB-610 [280] etc.

The “off-target” effects of TKI are expanding beyond small molecule inhibitors targeting FGFR and VEGFR pathways. In particular, crizotinib, a multi-kinase inhibitor targeting c-Met, ALK, and ROS1, exhibited a broad spectrum of such effects, including the down-regulation of ABCB1 and ABCG2 functional activity [258,281], inhibition of EMT [282], depletion of CSCs [283], and enhancement of radiation-induced DNA DSBs [284].

Similarly, imatinib, the non-selective v-Abl, c-Kit, and PDGFR exhibited broad spectrum of the “off-targeted” activities, including induction of DNA damage in the leukimea cells expressing the fusion protein [285], inactivation of the ATM/ATR signaling pathway after doxorubicin-induced DNA damage [286], decrease of ABCB1 and ABBC10 functional activity [287,288], inhibition of EMT [289], decrease in proliferation/differentiation of MSCs [290] and CSCs [291].

Collectively, antiangiogenic TKIs specifically targeting VEGFR and FGFR pathways demonstrated high clinical potencies against a broad spectrum of human malignancies and are currently used as a monotherapy regimen. Besides this, a growing body of evidence also suggests that these small-molecule inhibitors affect DNA repair pathways and EMT, modulate the activities of ABC transporters, and regulate the number of CSCs. These “off-target” effects of TKIs were described in detail above and summarized in Table 1.

Based on these effects, TKIs have also been shown as promising agents sensitizing tumors to conventional chemotherapies, which, in turn, open novel therapeutic avenues for combined cancer therapies, especially as second- or third-line treatments in patients who have progressed on modern chemotherapeutic regimens. In particular, anlotinib, a multi-kinase TKI targeting VEGFR, FGFR, and PDGFR, was used in combination with chemotherapy for NSCLC patients as a second- or third-line treatment and has proven to be effective and well-tolerated [297,298,299,300]. Similarly, apatinib, a VEGFR2 inhibitor, used in combination with docetaxel, was well tolerated and more effective when compared with docetaxel monotherapy for patients with NSCLC [301]. Clinical effectiveness of another mitotic-poisoning agent, docetaxel, was also improved in non-small-cell lung carcinoma (NSCLC) patients when used in combination with nintedanib, a non-selective inhibitor targeting all subtypes of VEGFRs, FGFRs, and PDGFRα and β, together with RET and FLT3 [302]. The strategy of using TKIs to potentiate the clinical efficacy of chemotherapies is not solely specific to FGFR and VEGFR inhibitors. It can extend beyond the inhibitors of these signaling pathways. In particular, lapatinib, a dual TKI that blocks HER1 and HER2, improved the clinical outcome of HER2-positive breast cancer patients when used in combination with paclitaxel [303]. Erlotinib, the EGFR inhibitor, potentiated the cytotoxic activity of gemcitabine and improved PFS and OS in pancreatic cancer [304,305], etc. Similarly, imatinib, a multi-kinase inhibitor targeting Bcr-Abl, Kit, and PDGFR kinases, and crizotinib, a potent inhibitor of ALK and c-MET, effectively impaired the function of several ABC transporters, inhibited EMT, and regulated the population of CSCs (as shown in Table 1).

## 18. Conclusions

Overall, TKIs exhibiting FGFR and VEGFR inhibitory activities demonstrated a significant therapeutic efficacy when used as a monotherapy or in combination with other TKIs and/or conventional chemotherapeutic agents, as well. The last one becomes especially important for therapy of MDR tumors lacking specific molecular targets and acquired chemo- and radioresistance via diverse mechanisms, as shown above. Indeed, several antiangiogenic TKIs targeting PDGFRs, VEGFRs, and FGFRs demonstrated clinical benefits for cancer patients when used in combination with chemotherapy, when compared to chemotherapy alone. Therefore, repurposing TKIs for cancer therapies by utilizing their broad spectrum of “off-target” effects, as illustrated above, and using them as adjuvants in chemotherapy, especially in drug-resistant tumors, may be a promising therapeutic strategy to overcome MDR in cancer and develop effective and low-toxic combination therapy. However, maximal inhibition of MDR and its minimal pharmacokinetic interaction with the chemotherapeutic drugs may be a challenge. The other challenge lies in effectively integrating RTKIs, which impact the tumor’s vasculature and potentially impair drug delivery, with cytotoxic agents. Additionally, the most effective TKIs would be the agents with maximal inhibitory activities against their primary molecular target and exhibiting low affinity to ABC transporters to prevent their extensive efflux from cancer cells. Future research should also focus on understanding the complexity of the tumor microenvironment and drug delivery mechanisms to optimize RTKIs/chemotherapy combinations and achieve better clinical outcomes.

## Figures and Tables

**Table 1:** Non-targeted (i.e., “off-target”) effects of receptor tyrosine kinase inhibitors (RTKIs).

Drug	Primary Target	Non-Targeted (i.e., “Off-Target”) Effects			
		DNA Damage Response (DDR)	ABC Transporters	Epithelial-to-Mesenchymal Transition (EMT) or Mesenchymal-to-Epithelial Transition (MET)	Cancer Stem Cells (CSCs)
Infigratinib (BGJ398)	pan-FGFR	Inhibited HR-mediated DNA repair [159]	↓ ABCB1 functional activity [167]	Inhibited EMT (↓ Vimentin and Slug) [292]	↓ ALDH^high^CD44^high^ cells [175] ↓ ALDH7A1 and OCT4 [177]
Fexagratinib (AZD4547)	FGFR1, FGFR2, and FGFR3	Inhibited PTEN-mediated DNA repair [161]	ND	Induced MET [179]	↓ ALDH-positive cells [176] Reduced the number of CSCs [179]
Erdafitinib	pan-FGFR	Induced ROS-mediated DNA damage [163]	↓ ABCB1 functional activity [165,166]	ND	ND
Pemigatinib	FGFR1, FGFR2, FGFR3, and FGFR4	Decreased irradiation-mediated DDR [162]	↓ ABCB1 functional activity [169]	ND	Decreased CSC survival and proliferation [162]
Ponatinib	BCR-ABL, FGFR1, VEGFR2, PDGFRα, Abl, RET, and Src	ND	↓ ABCB1 and ABCG2 expression and functional activity [185] ↓ MRP7 expression and functional activity [186]	Inhibited EMT (↑ E-cadherin;↓ Vimentin, p-Smad3) [187]	↑ *NANOG* and *SOX2* [188] ↓ CSCs [189]
Dovitinib (TKI-258)	KIT, FGFR1-3, VEGFR1-3, PDGFR A/B/, RET, and FLT-3	Targeted topoisomer-ase I and topoisomer-ase II [194] Inhibited BER and MGMT [193]	↓ ABCB1 and ABCG2 functional activity [191]	Inhibited EMT [196]	Inhibited CSC-like protein Lin28 and its target HMGA2 [193]
Pazopanib (GW786034)	c-KIT, FGFR, PDGFR, and VEGFR	Induced DNA damage and cellular senescence [206]	↓ ABCB1 and ABCG2 functional activity [198]	Inhibited EMT (↓ VE-cadherin) [207]	ND
PD173074	FGFR1 and VEGFR	ND	↓ ABCB1 and ABCC10 functional activity [208,209]	Induced MET (↑ E-cadherin, ↓ Snail1) [173]	ND
Axitinib	VEGFR1, VEGFR2, VEGFR3, PDGFRβ, and c-Kit	Induced senescence and/or mitotic catastrophe [293,294,295] Activated DDR (↑ γ-H2AX, Chk1, p21) and G2/M arrest [293,295]	↓ ABCG2 functional activity [235]	Inhibited EMT (↓ Vimentin) [238]	Targeted CSC-like cells [235] Decrease CSC proliferation [239]
Regorafenib	VEGFR1-2, TIE-2, PDGFRβ, FGFR1, RET, c-KIT, BRAF, and c-RAF/Raf-1	Inhibited radiation-induced DDR [248] Induced DNA damage and decreased repair ability [249]	↓ ABCB1 functional activity [243] ↓ ABCG2 functional activity [245]	Inhibited EMT (↑ E-cadherin, ↓ SNAI2 and Vimentin) [252]	Decreased the stemness phenotypes [254]
Cabozantinib (XL184)	VEGFR2 and MET	ND	↓ ABCB1 functional activity [260] ↓ ABCG2 functional activity [257,258,259]	Inhibited EMT [262]	Downregulated CD133 (↓ SOX2) [261]
Sunitinib	PDGFR, VEGFR, Kit, and FLT-3	Attenuated HR-mediated DNA repair [265] Induced non-repaired DNA DSBs and G1/S cell cycle arrest [266] Induced chromosome instability, DNA damage, and p53-dependent apoptosis [267] Enhanced radiation-induced DNA DSBs and induced G2/M arrest [268]	↓ ABCB1 functional activity [264] ↓ ABCG2 functional activity [263,264]	ND	Downregulated ALDHhigh CSC-like cells [270] Decreased CSC proliferation [239] Disrupted CSC s differentiation into endothelial cells in vitro and vasculo-genesis induced by CSCs in vivo [273]
Motesanib (AMG-706)	VEGFR1, VEGFR2, VEGFR3, Kit, PDGFR, and Ret	ND	↓ ABCB1 and ABCG2 functional activity [278]	ND	ND
Telatinib (BAY 57-9352)	VEGFR2/3, c-Kit, and PDGFRα	ND	↓ ABCG2 functional activity [279]	ND	Targeted FSL3, overexpressed in colorectal CSCs [296]
SKLB-610	VEGFR2, FGFR2, and PDGFR	ND	↓ ABCG2 functional activity [280]	ND	ND
Crizotinib	c-Met, ALK, and ROS1	Enhanced radiation-induced DNA DSBs [284]	↓ ABCB1 and ABCG2 functional activity [258,281]	Inhibited EMT (↑ E-cadherin; ↓ Vimentin) [282]	Targeted CSCs [283]
Imatinib	v-Abl, c-Kit, and PDGFR	Induced DNA alkali-labile sites [285] Inhibited the S–G2–M transition after Adriamycin exposure and inactivated ATM/ATR signaling pathway [286]	↓ ABCB1 and ABBC10 functional activity [287,288]	Inhibited EMT (↑ E-cadherin; ↓ Fibronectin, SNAI2) by modulating the Notch signaling pathway [289]	Decreased proliferation of human MSCs [290] Inhibited differentiation of CSCs [291]

ND—no data; HR—homology recombination; ROS—reactive oxygen species; BER—base excision repair; MGMT—O6 -methylguanine-DNA-methyltransferase; HMGA2—high-mobility group protein A2; VE-cadherin—vascular endothelial cadherin; DSBs—double-strand breaks; MSCs—mesenchymal stem cells. ↑: increased expression/activity; ↓: decreased expression/activity.

## Data Availability

The original contributions presented in this study are included in this article. Further inquiries can be directed to the corresponding author.
